# Mr. Young, in Reply to Dr. Adams

**Published:** 1806-11

**Authors:** Samuel Young

**Affiliations:** No. 22, Welbeck Street


### To Dr. ADAMS,

Sir,

THE full conviction that a proper knowledge only of its nature can lead to any thing like a successful treatment in cancer, induced me to address you on the subject.* The same motive of course actuates my reply. I shall follow (as much as is consistent with the question) the example you set me in the brevity of your answer.† I have still however to claim the indulgence of unreserved investigation; my apology you already have in the evident connexion in the present case, betwixt theory and practice.

To the end then of practical improvement, I shall unreservedly reply to your answer, and trust it will soon be made apparent how incapable the hydatid hypothesis is of defence, even from the ablest advocate.

In way of recapitulation let me first observe, that the letter I had the honor of addressing to you, ended with *seven* articles of objection, not to *one* of which is there a *single* attempt at refutation in your answer; I therefore conclude them to be unanswerable objections. The three first of these articles I shall keep particularly in view.

I have also to premise, that *now* one can only consider an hypothesis, which gave to the cancerous disease, an hydatid or animalcular existence; and therefore the observation you make, Sir, in your answer to me, "that you have never yet entered into the question, whether hydatids found in the liver have a life and economy independent of the animal in which they are found, excepting as they draw their nourishment from it, is a mental reservation entirely your own. I appeal to all that ever read the hydatid hypothesis, nay I appeal to the very correspondence itself, whether an animalcular existence was not distinctly meant and understood. What does Dr. Baillie say? "Respecting the propriety of the term *cancerous hydatid,* I have only to remark, that if you can prove the cyst in a schirrous structure to possess a life peculiar or proper to themselves, like the hydatids found in the liver, there would seem to be no impropriety in the term. But if they possess merely a living principle, similar to that of the ordinary diseased structures in an animal body, I should think it better for you to adhere to the name of cyst." This (considering the Doctor's *ineffable indifference)* is certainly

* Vide Med. Journ. No. 87.

† Vide No. 90.

strong strong and pointed language. What are the words of Mr. Cline?—"Hydatids are no more a part of the body in which they are found, than worms in the intestinal canal." What is this, but in express opposition to the *animalcular nature* you give to cancer? Nay, such an animalcular impression has the hypothesis made, that in a late publication from the sister kingdom, able analogies (I am told) are formed between the œconomy of cancer and those of truffles and morels!

It was at the time of the *original* correspondence that an objection to such impressions should have been taken. It was *then* most assuredly the time distinctly to state any modifications of sense or meaning. At *this advanced stage* of the question it is impossible to recognize such, but *as formal recantations*.

I believe there is scarce a doubt in any one's mind, but what the human hydatid, from its strong analogies to those found in the brain of sheep, is a species of animalculæ. The paper of Dr. Hunter's, which you prefixed to your correspondence, evidently goes in proof of the fact, and was as evidently placed there to shew the similarity. Therefore I do not see what possible difference is gained by the following modification, in answer to my letter. "There is the same reason to admit a separate existence in one species of cancerous breast, as in the hydatids of the liver; and that as the human mamma is found a convenient nidus for the common hydatid, there is a presumption that other substances found in it, may have a similar œconomy." A thing either is, or is not. The only difference I can possibly form by the distinction as it here stands, is, that instead of attributing *directly* an animalcular or distinct nature to cancer, it is now done *indirectly*, by altering the term into "similar economy," which, after all, amounts to the same thing; for a similar œconomy certainly implies a precise analogy.

Without once noticing the objections I urge in proof against motion in a part, *alone* being sufficient to prove the question, as deduced from the contractility evinced in the cells of cancerous fat; you still repeat, (without any attempt to shew how such an evidence of life necessarily infers a distinct œconomy) that "the only proof we require of life is motion, independant of elasticity." What are we to infer from this, but that motion (according to your ideas) necessarily infers a distinct œconomy? For you never say a word (otherwise) of the independancy of the fatty cells from the surrounding parts: You have never yet endeavoured endeavoured to shew, in answer to Mr. Cline's observation, that these cells are as distinct as worms in the intestinal canal, and which he pointedly tells you the common hydatid is, from whence you draw your reasonings, and rest the conclusion that in such cells a similar œconomy exists.

You do indeed tell me, on the score of distinction, that nothing is so easy as to observe the difference between such morbid changes and healthy structure. Surely, Sir, no one could ever doubt it; for if a difference had not existed, how could you ever have noticed it? But, Sir, my question went to ask, whether you could prove these cells to be *independent* of the surrounding parts? Not, whether they were *different* from the neighbouring parts! Diseased fat we all know to be very different to healthy adipose; but it must be proved of the former (though its cells may have acquired a visible contractility) that it is totally independant of the latter, as worms are of the intestines, before one can allow an hydatid existence, or what is the same thing, "a similar œconomy."

Since you have rested so much on the contractility of the fatty tunics, and since I have so fully denied such evidence to be competent, I think it but right shortly to enlarge on the faculty of motion as connected with life and structure.

It is true there cannot be more satisfactory evidence of life, than that of contraction in a part on the application of stimuli: but non-contractility is by no means a proof of the absence of life. An egg, without any apparent faculty of motion, affords equally satisfactory evidence of the possession of life. Nor are we to deny the faculty of motion to such bodies, because it is not evident to our senses. Visible motion in living bodies would appear to attend a more exquisite possession of irritability or principle of life; and this not necessarily depending on any particular structure, though it would seem to be more commonly found in such as are termed muscular. There is no reason whatever (as far as structure is concerned) why the substance of an egg should not possess as great a faculty of motion as a bundle of muscular fibres. The pulpy or jelly-like structure of many fresh water polypi is equally tender with that of the egg, equally destitute of fibrous texture; yet they possess the faculty of motion in a very remarkable degree; nor is there an animal more irritable.

Since therefore the faculty of motion would appear exclusively to arise out of life, and not to depend on any particular structure; we must of necessity allow the possession of such faculty to every part that affords us the same or similar evidence of life with the egg. The degree may be very low, but because it is not evident to our gross perceptions, are we to deny the possession of the faculty altogether? One might as well, watching the merry revolutions of a second hand round the dial of a time piece, deny all motion to its companions the minute and the hour hands. Such conclusions would be something like the countryman who laughed at the powers of electricity, from having only experienced the tickling sensation of the spark; till a well charged jar gave him a *striking instance*, and cured him of his heresy.

We know likewise, that this faculty of motion may be highly increased or diminished, or even partially suspended, in the self same structure.

Experiments on animals that have breathed oxygen gas for some time, demonstrate a very considerable increase of this faculty. When opened immediately after being drowned, their intestines have been thrown into twenty fold the motion upon the application of stimuli, than those of other animals which had only breathed commonly.

The hearts and intestines of other animals that had been exposed to a greater dilution of oxygen, either by an admixture of azotic or hydrogen gas, scarcely afforded any evidence of motion: in some, none whatever could be perceived. Now, in all these it is very evident the same faculty must have existed for a given time, but in different degrees; not unaptly exemplified by the different relations of motion in the three hands of a second watch.

Following up the stimulus on the most irritable parts, no motion in a short time can be perceived; but are we to conclude, that all faculty of motion ceases precisely at that instant when it ceases to be evident to our senses? That indeed would be to say, that nothing exists beyond the range of our perceptions!

But facts do not stop here. A curious evidence is still afforded by the same experiment; which is, that after all motion has apparently ceased in the part for some time, yet by a close attention, and repeating the stimulus at short intervals, it will again become visible. And this is perfectly agreeable to the laws of irritability. So that in fact, with regard to visible motion, you have three points of evidence in the self-same structure, viz. its existence; its extinction; and its revival.

Is it not therefore fair to conclude, that there is every reason reason to allow a certain degree of the faculty of motion to every body that possesses life; since it is evident that on that principle alone it depends, unconstrained by structure; (so far, at least, as we find more motion in a mere pulp than the best marked muscle) and since there is no reason whatever to deny the existence of the faculty, because its effect may not be evident to our senses?

There is no question, I believe, but what adipose membrane possesses a certain degree of life; and since the faculty of motion, uninfluenced by structure, would appear necessarily the common attendant of what is termed irritability, or life; and since such faculty may be increased, and diminished (and that too evident even to our own perceptions) to an almost unlimited variety in the same part; where is the difficulty of allowing a certain low degree of such faculty to exist in adipose, and which might become faintly visible (as you instance in the cancerous cells) by an accession of morbid irritability?

If this reasoning however be not correct, the motion in the cells you have discovered cannot support the conclusion of a distinct existence in them; and which you so evidently wish to infer, when you say, "the only proof we require of life is motion." In the common adipose cellular membrane we have nothing like motion. It is true, in *visibly* possessing such faculty (I now speak upon your authority, not having seen it myself) they differ from *healthy* adipose, but in every other respect, as far as *disease* goes, they are under the *common connexion* of *common* or *original* structure; and therefore, (wanting that essential characteristic of a separate œconomy, viz. a perfect state of independancy from the surrounding parts) contractility alone cannot prove an animalcular nature in them. Or else the same, or similar œconomies, must of necessity be given to the hearts and intestines of the animals just mentioned; since they afforded precisely the same evidence, and that too in a much more unequivocal manner.

You say, we never see a series of abscesses resembling each other in size and form, attached laterally to each other, and filled with a secretion, which is found in every part of a healthy body.

The only direct mention I made of abscess was to account for the different arrangements of the vessels on the surfaces of diseased cysts. Respecting these *laterally* attached cells (which I contend are ab origine so connected in the healthy membrane) I said no extraordinary inference could justly be drawn from the evidence of their altered appearance, when it was recollected to what a complicated morbid progress they had been exposed; and particularly since condensation, and lateral obliteration in such cellular structure are the familiar results of common abscess. I see no earthly reason why abscesses should be found linked laterally together, because these cells are. If however we come to the strictness of the thing, lateral connexion is by no means a necessary, or even usual mode of hydatid growth, and therefore can prove no peculiarity to the circumstance of small cells being found laterally connected to each other. One might indeed begin to talk of peculiar œconomies, if instead of such uniform lateral connexion, these cells had ever been found detached, or some irregularly adhering in clusters, evincing different degrees of growth. It is on the contrary their very regularity and uniform lateral attachments, which connect them in the mind with original structure.

These considerations I offer in further illustration of what may be found in my former letter in support of the three first objections I took against the hydatid hypothesis; and which (what I now think amounts to a demonstration) your answer has irresistably established; so far, as the solitary fact of the contraction of the cells is not by any shadow of pretence shewn necessarily to infer an animalcular œconomy; nor any thing proved like an animal independancy, by any evidence whatever of such cells being a subsequent production, *wholly* unconnected with original structure.

I have to lament, that among all the objections to be found in my former letter against the hypothesis, you have only thought it necessary to notice two of them in your answer; and which indeed are the very two that I answered myself. I allude to the constant *lateral* mode of growth of the cells; and the very common circumstance of finding schirri in *different points* of the breast, with *healthy* structure intervening; the possibility of which, I admitted might be consistent with *hydatid growth;* although in forming a theory from *analogy,* one would wish to have probabilities as strong, and as little left to conjecture as possible. For certainly the formation of hydatids at certain distances from each other, at the same period of time, is not so frequent as that of schirri; nor can we account (animalcularly) for the frequency of such formation in the latter, by an escape of the young in a fluid, which we know is a common occurrence in the instance of the former.

The

The remainder part of the Answer I cannot but consider as an acquiescence to my former objections and reasonings. The theory of a disease is the concording result of broad historical facts; particular exceptions may come under the cognizance of nice practical distinctions, but they never can form a general doctrine. That indeed would be to call a romance a history, because it is grounded on some historical fact. How then can we call *that* a theory of cancer, the ground work of which we are told can only be found "in one species of cancerous breast?" Or, how can that be supposed to be a history even of that particular species, which is wholly founded on presumptive analogy, drawn from a solitary inconclusive fact. With such materials the most brilliant capacity must be obscure; the truth of which nothing can more forcibly speak than the following sentence.

"If I am not intelligible I must despair of being so, and trust to the industry of others in recording what they discover, or to future opportunities of demonstrating in the recent subject, what I find in vain to describe in words." Here the hypothesis falls. And I rejoice in full confidence, for the sake of humanity, (so far as it adds to the adventitious obduracy of a disease, a systematic inveteracy which excludes all hope of relief) that, if it waits the evidence of facts, it will rise no more.

It is however to be confessed, that although every thing but utility may arise directly from the hydatid hypothesis itself, yet the surgery of cancer no doubt will be advantaged by the example you have set in liberally coming forward in an inquiry, which it is to be lamented has hitherto been denied the common share of common attention. For myself in particular, Sir, I have to acknowledge the temper you have shewn in the present investigation. Which still persisting to urge the necessity of future inquiry, even at the expence of your own opinions, affords a striking contrast to those, who reaping the advantages of unthought of practice, and having talents, yet are so dead to the welfare of that very public which so abundantly affords them support—so selfishly fearful of exposing themselves to the *indignity* of refutation, as even to make it a common boast, that they never *reason on Medicine*; and absolutely chuckle in the ease and security of a *criminal indifference.*

I remain, &c.

SAMUEL YOUNG.

